# Legionnaires’ disease in the EU/EEA*: increasing trend from 2017 to 2019

**DOI:** 10.2807/1560-7917.ES.2023.28.11.2200114

**Published:** 2023-03-16

**Authors:** Jonas Samuelsson, Lara Payne Hallström, Gaetano Marrone, Joana Gomes Dias

**Affiliations:** 1 European Centre for Disease Prevention and Control, Stockholm, Sweden

**Keywords:** Legionella, Legionnaires’ disease, food-and-waterborne diseases, surveillance, time- series, trends

## Abstract

**Background:**

The burden of Legionnaires’ disease (LD) in the European Union/European Economic Area (EU/EEA) has increased during the last decade, with notification rates increasing from 1.2 to 1.4/100,000 population in 2012–16, to 1.8–2.2 within 2017–19.

**Aim:**

To measure weekly excess cases during 2017–19 based on previous trends and determine whether a significant change in trend occurred, and to examine any differences in age, sex or level of imported infections.

**Methods:**

We collated 2012–19 annual surveillance data from The European Surveillance System (TESSy) reported by EU/EEA countries. A retrospective prediction by a dynamic regression model was created from 2012–16 data to assess excess cases in 2017–19. Interrupted time series (ITS) analysis was performed to determine if a significant change in trend occurred in 2017–19 compared with the previous 5 years.

**Results:**

We found a 33.9% increase in cases in 2017–19 compared with the number predicted. The ITS also found a significant trend increase in 2017–19 compared with 2012–16. A significant trend increase was observed from 2017 most strongly among older age groups (> 60 years) and non-imported cases.

**Conclusion:**

Our study showed a significant increasing trend in LD cases in the EU/EEA during 2017–19 compared with the previous 5 years. The distribution of cases per week suggests an overall amplification of the seasonal trends. These findings underscore that LD continues to be an infectious disease of public health concern in the EU/EEA, warranting further research into determinants of the increase.

**Key public health message**

**What did you want to address in this study?**

Legionnaires’ disease is a severe, sometimes fatal pneumonia caused by inhalation of water aerosols containing *Legionella* bacteria. We aimed to assess if the observed increase in Legionnaires’ disease cases in the EU/EEA in 2017–19 was significantly different than in the previous 5 years. The study looked at the occurrence and trend in cases according to age group, sex and level of imported infections.

**What have we learnt from this study?**

This study showed a significant increase and change in trend in notifications of Legionnaires’ disease cases in the EU/EEA during 2017–19, with an amplified seasonality and excess in all study age groups, males and females, as well as non-imported cases. However, among age groups, the strongest significant trend from 2017 was observed in age groups > 60 years.

**What are the implications of your findings for public health?**

Our study shows that Legionnaires’ disease continues to be a disease of public health concern in the EU/EEA, with increasing numbers of cases reported under 2017–19. *Legionella* prevention and monitoring of disease trends remain important.

## Introduction

Legionnaires’ disease (LD) is a severe pneumonia caused by inhaling aerosols containing *Legionella* bacteria. The bacteria are commonly found in fresh water around the world and in engineered water systems with suitable nutrient and temperature conditions for growth. People aged 50 years and older are at a higher risk of becoming ill after exposure, and the risk is higher among males than females. Further risk factors include chronic lung disease, smoking and being immunocompromised. Timely detection is important for appropriate antibiotic treatment and facilitating recovery [1].

Legionnaires’ disease surveillance data in the European Union/European Economic Area (EU/EEA) is collated at the European Centre for Disease Prevention and Control (ECDC) through annual reporting by EU/EEA countries through the European Legionnaires’ disease Surveillance Network (ELDSNet) to The European Surveillance System (TESSy), providing an estimate of the occurrence of this disease in Europe. The annual LD notification rate in the EU/EEA slowly increased between 2012 and 2016, from 1.2 to 1.4 cases per 100,000 population [2,3].

In 2017, the notification rates increased to 1.8 per 100,000 (9,260 cases), followed by an even higher rate of 2.2 per 100,000 population in 2018 (11,403 cases) and 2019 (11,298 cases) [3]. Since 2011, four countries – Italy, France, Germany and Spain – consistently report the majority of LD cases annually in the EU/EEA, and age-standardised rates in Denmark, Italy and Slovenia are highest [4]. There were no changes in the EU/EEA surveillance case definition for LD during 2012–19, nor in the surveillance system. No reported outbreak events could explain such notification rate increases. A change in disease trend was hypothesised.

Using two different time-series analysis methods we aimed to (i) analyse whether the number of observed cases in the EU/EEA during the period 2017–19 was statistically significantly higher compared with the period 2012–16 and (ii) estimate any significant change (increase) in trend in 2017–19 compared with 2012–16. Moreover, we aimed to assess if there were differences according to age, sex or importation of infection.

## Methods

### Data collection

A dataset containing data on 64,559 LD cases reported by EU/EEA countries to ECDC for the years 2012–19 was extracted from TESSy, with the latest annual data available, in November 2022. We excluded cases reported by Croatia and Iceland since annual data from these countries were not available for all years, and by Bulgaria, Germany, and Luxembourg because of a lack of a weekly date variable or information to determine the likely importation status of infection. These five countries contributed to 13.4% of the initial data (12.9% by Germany and 0.5% by the other four), resulting in 8,651 cases excluded from 2012 to 2019. The percentage of cases for which these five countries with excluded data comprised each year during 2012–19 remained stable at around 13% (range: 10.9%–14.3%).

The study period ranged from week 1 in 2012 to week 52 in 2019. When date of disease onset was not available (n = 1,703 corresponding to 3.1% of cases), we used the country reported variable ‘Dateusedforstatistics’ [5] as proxy. This has been shown to be close, with a mean difference of 2.4 days (standard deviation: 18.4 days) in the dataset for the 54,255 cases for which both dates are reported.

This resulted in a dataset for the analysis of 55,821 cases from 25 countries: Austria, Belgium, Cyprus, Czechia, Denmark, Estonia, Finland, France, Greece, Hungary, Ireland, Italy, Latvia, Lithuania, Malta, the Netherlands, Norway, Poland, Portugal, Romania, Slovakia, Slovenia, Spain, Sweden and the United Kingdom.

The variables analysed in this study were: age group, sex and imported. The variable ‘age group’ was created by stratifying reported case age into the following categories: < 40, 40–49, 50–59, 60–69, 70–79 and ≥ 80. The variable ‘imported’ was created for this study based on the TESSy variables ‘Imported’ and 'Setting’ [5] that indicate whether the case may have a probable ‘setting of infection’ such as travel abroad, or a history of travel abroad under their incubation period (2–10 days before date of symptom onset).

### Statistical analysis

The epidemiology of LD in 2012–19 was described overall and stratified by age, sex and importation of infection. Age-group standardisation was not considered relevant for the aims of the study, as the intention was a description of magnitude of excess for each age group, not a relative comparison.

A retrospective prediction of LD cases in 2017–19 was estimated based on 2012–16 log transformed observed data *log(Yt)* using harmonic regression models with Fourier terms (k) and autoregressive integrated moving average (ARIMA) errors [6]. The model was fitted using a two-step approach inspired by De Livera, Hyndman and Snyder [7]: first, the number of harmonics used for the seasonal component was selected by finding the lowest value of the Akaike information criterion (AIC). Then, ARIMA components were added by differencing (d) the data, adding a number of autoregressive (p) and moving average (q) terms, as well as a trend component 1 if still needed after differencing the data, i.e. by computing a new time series consisting of the differences between consecutive observations to make the time series stationary and reducing trend and seasonality:


$$log\left(Yt\right)'=ARIMA\left(p,d,q\right)+Fourier\left(k\right)+1$$

Where:


$$log{\left(Yt\right)}^{'}=\log\left(yt\right)-log\left(yt-1\right)$$

Both selections were carried out by an algorithm in the fable package in R [8]. Whether the model was differenced, the number of autoregressive, moving average, and Fourier terms that were included and whether a slope needed to be added was decided by which combination gave the lower AIC. This was to find the model that best fits the 2012–16 data. For building the models, we have relied on the suggestions by Hyndman and Athanasopoulos [6].

We then created a prediction (forecast) of the weekly LD cases for the next 3 years. This gave us a predicted number of cases for each week during 2017–19, along with 80% and 95% prediction intervals (PI). For the cases above prediction, for each year, we subtracted the predicted cases from the observed ones to measure any excess. We also calculated, for each year, the number of cases above and below the 80% and 95% prediction interval and the number of weeks when at least one case above or below the 80% and 95% prediction interval was observed.

Interrupted time series (ITS) analysis was performed to determine if there had been a significant change in trend of LD incidence in 2017–19 compared with the previous 5 years. ITS is used to compare the trends observed over two different time periods and to assess significant changes in point estimates (short-term effects) and trends (long-term effects) of an outcome of interest [9]. In the ITS, we compared the period 2012–16 with 2017–19 to test the hypothesis that the trend had changed significantly. A linear regression was fitted using the calendar week *T* as time variable, and a dummy variable *X* equal to 0 for the period before 2017 and equal to 1 from 2017. In this way, β_3_ represents the change in trend observed in the period 2017–2019 (when the trend is given by β_1_ + β_3_) compared with the trend observed in the period 2012–16 (when the trend is given by β_1_). We used seasonal and trend decomposition by Loess (STL) to remove the seasonal component while estimating the trend [6]. A 1-week lag of the seasonally adjusted cases (β_4_) was added to produce a better model fit as the weekly number of cases were autocorrelated.

The final model had the following equation:


$$E\left(Y_{t}\right)=\beta_{0}+\beta_{1}T+\beta_{2}X+\beta_{3}TX+\beta_{4}Y_{t-1}$$

A smoothed linear graph was then created to visualise the changes in point estimates and trend.

The assumptions of constant seasonality, stationary data, no autocorrelation in the errors, and normally distributed errors were verified by plotting the residuals over time, the autocorrelation functions (ACF) and the histograms.

All analyses were conducted using R software (version 4.0.5) [8]. For the time series analysis, we used the tidyverts packages: tsibble, fable, fabletools, and feasts [10]. Scripts with the R code used to produce the results presented in this publication are available on GitHub at: https://github.com/EU-ECDC/LegionnairesDiseaseInEUEEA.

P values less than 0.05 were considered statistically significant.

### Sensitivity analysis

An unusual outbreak of LD in Portugal in November 2014 [11] contributed to 80.2% of 363 cases in an unprecedented observation in weeks 45 of 2014 in the TESSy dataset. We built prediction models with and without the 291 outbreak cases reported to determine its impact on the predictions.

## Results

### Descriptive analysis

**Figure 1:**
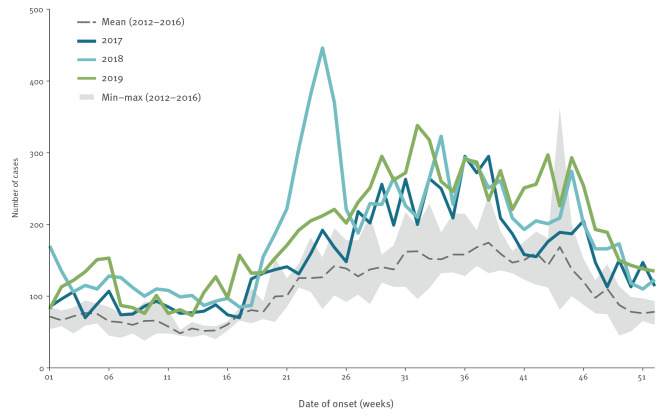
Reported Legionnaires’ disease cases by week of onset, 25 EU/EEA countries, 2017–2019 vs 2012–2016 comparative range (n = 55,821)

The weekly distribution of cases for each year within 2017–19 and the comparative distribution for the period 2012–16 is shown in Figure 1. It indicates the more extreme variation in weekly case numbers observed during 2017–19. This is mostly visible during the summer and early autumn (June to September) which is the usual high season for the disease in the EU/EEA.

EU/EEA: European Union/European Economic Area.

EU/EEA countries with data for 2012–19 included in this analysis are: Austria, Belgium, Cyprus, Czechia, Denmark, Estonia, Finland, France, Greece, Hungary, Ireland, Italy, Latvia, Lithuania, Malta, the Netherlands, Norway, Poland, Portugal, Romania, Slovakia, Slovenia, Spain, Sweden and the United Kingdom.

Of the 55,821 LD cases during the 2012–19 period presented in Table 1, 49.3% (27,530 cases) were observed during 2017–19. Each of these 3 years had more cases annually than any other year in the study period. The weekly interquartile range (IQR) of case numbers doubled between 2012 and 2019.

**Table 1:** Annual reported number of Legionnaires’ disease cases by age, sex and likely importation of infection, 25 EU/EEA countries, 2012–2019 (n = 55,821)

Reported cases	2012		2013		2014		2015		2016		2017		2018		2019		Total	
	n = 5,173		n = 5,023		n = 6,012		n = 6,036		n = 6,047		n = 7,935		n = 9,854		n = 9,741		n = 55,821	
	n	%	n	%	n	%	n	%	n	%	n	%	n	%	n	%	n	%
Cases per week																		
Median cases per week (IQR)	100	60–138	94	65–125	102	70–153	102	75–146	115	80–152	147	92–200	188	112–228	188	129–252	122	80–167
Age (years)																		
Median (IQR)	63	51–74	64	52–74	62	52–73	64	52–64	63	52–74	65	53–75	64	53–75	65	54–75	64	53–75
< 40	352	6.8	332	6.6	349	5.8	380	6.3	351	5.8	444	5.6	562	5.7	536	5.5	3,293	5.9
40–49	714	13.8	598	11.9	824	13.7	718	11.9	720	11.9	841	10.6	1,074	10.9	974	10.0	6,475	11.6
50–59	1,138	22.0	1,060	21.1	1,395	23.2	1,346	22.3	1,373	22.7	1,706	21.5	2,128	21.6	2,007	20.6	12,169	21.8
60–69	1,205	23.3	1,226	24.4	1,383	23.0	1,449	24.0	1,469	24.3	1,849	23.3	2,326	23.6	2,367	24.3	13,285	23.8
70–79	941	18.2	1,020	20.3	1,160	19.3	1,183	19.6	1,155	19.1	1,650	20.8	1,991	20.2	2,085	21.4	11,220	20.1
≥ 80	781	15.1	779	15.5	896	14.9	954	15.8	968	16.0	1,444	18.2	1,764	17.9	1,763	18.1	9,322	16.7
Unknown	41	0.8	10	0.2	6	0.1	6	0.1	12	0.2	0	0	10	0.1	10	0.1	56	0.1
Sex																		
Male	3,673	71.0	3,546	70.6	4,250	70.7	4,280	70.9	4,251	70.3	5,562	70.1	6,947	70.5	6,789	69.7	39,410	70.6
Female	1,500	29.0	1,472	29.3	1,683	28.0	1,738	28.8	1,796	29.7	2,357	29.7	2,907	29.5	2,942	30.2	16,411	29.4
Unknown	0	0	5	0.1	78	1.3	18	0.3	0	0	16	0.2	0	0	10	0.1	0	0
Imported from abroad																		
Yes	574	11.1	522	10.4	619	10.3	748	12.4	677	11.2	889	11.2	877	8.9	896	9.2	5,917	10.6
No	4,490	86.8	4,385	87.3	5,339	88.8	5,251	87.0	5,303	87.7	6,896	86.9	8,819	89.5	8,611	88.4	49,904	89.4
Unknown	109	2.1	116	2.3	54	0.9	36	0.6	67	1.1	151	1.9	158	1.6	234	2.4	0	0

EU/EEA: European Union/European Economic Area; IQR: interquartile range.

EU/EEA countries with data for 2012–19 included in this analysis are: Austria, Belgium, Cyprus, Czechia, Denmark, Estonia, Finland, France, Greece, Hungary, Ireland, Italy, Latvia, Lithuania, Malta, the Netherlands, Norway, Poland, Portugal, Romania, Slovakia, Slovenia, Spain, Sweden and the United Kingdom.

No change in median age was observed over 2012–19 but there was a lower proportion of cases aged under 50 years and higher proportion of cases ≥ 80 years in 2017–19 compared with 2012–16. Each year, 8.9–11.2% of cases were likely to be imported from abroad and around 70% of cases were male.

### Retrospective prediction

**Figure 2:**
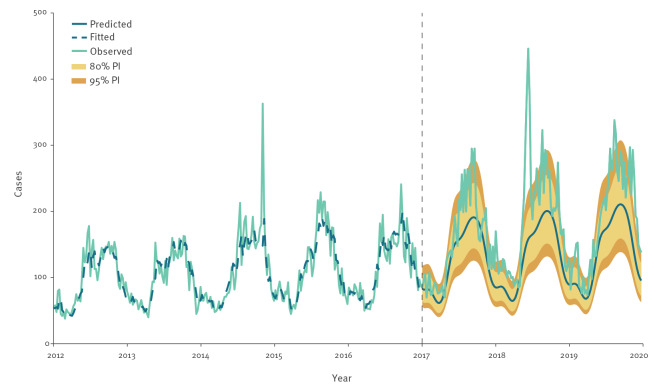
Weekly reported number of Legionnaires’ disease cases by date of onset, 25 EU/EEA countries, 2012–2019 (n = 55,821) showing retrospective prediction for 2017–2019 based on a 2012–2016 fitted model

As presented in Figure 2 and Table 2, the number of cases observed annually 2017–19 was higher than expected based on 2012–16 observations. Overall, for 2017–19, there was a 33.9% increase in cases compared with the expected levels. A total of 2,820 cases above the 80% PI and 1,287 cases above the 95% PI were observed, for a total count of 85 weeks and 39 weeks, respectively, under the 3-year period. Excess cases occurred throughout the 3 years but mostly following the usual seasonality of peaks in the summer and early autumn. The year 2017 had the smallest increase in cases above the 80% PI (n = 406) and the 95% PI (n = 88). The year 2018 had the largest increase above the 80% PI (n = 1,456) and above the 95% PI (n = 838), mostly because of the spike of cases observed in June.

EU/EEA: European Union/European Economic Area; PI: prediction interval.

EU/EEA countries with data for 2012–19 included in this analysis are: Austria, Belgium, Cyprus, Czechia, Denmark, Estonia, Finland, France, Greece, Hungary, Ireland, Italy, Latvia, Lithuania, Malta, the Netherlands, Norway, Poland, Portugal, Romania, Slovakia, Slovenia, Spain, Sweden and the United Kingdom.

Akaike information criterion (AIC): −126.195. Residual analysis plots are available in Supplementary Material S23.

**Table 2:** Reported, predicted and excess Legionnaires’ disease cases in 2017–2019 based on 2012–2016 weekly retrospective prediction models, total and by year, age group, sex, and likely importation of infection, 25 EU/EEA countries (n = 27,530 cases)

Year	Model	Observed cases (O)	Predicted cases (P)	Above prediction level		Above 80% PI		Above 95% PI		Under 80% PI		Under 95% PI	
				Cases (n) (O−P)	Percentage (%)	Cases (n)	No. weeks	Cases (n)	No. weeks	Cases (n)	No. weeks	Cases (n)	No. weeks
2017	Total	7,935	6,525	1,410	21.6	409	19	88	7	0	0	0	0
2018	Total	9,854	6,849	3,005	43.9	1,456	35	838	19	0	0	0	0
2019	Total	9,741	7,193	2,548	35.4	955	31	361	13	0	0	0	0
2017 –19	Total	27,530	20,566	6,964	33.9	2,820	85	1,287	39	0	0	0	0
	Age group (years)												
	< 40	1,544	1,062	482	45.4	170	58	47	16	< 5	4	0	0
	40–49	2,891	2,148	743	34.6	220	37	92	10	0	2	0	0
	50–59	5,840	4,875	965	19.8	321	32	114	9	< 5	3	0	0
	60–69	6,533	4,939	1,594	32.3	543	55	197	22	11	3	< 5	1
	70–79	5,736	4,141	1,595	38.5	599	75	236	35	< 5	2	0	0
	≥ 80	4,965	3,377	1,588	47.0	534	81	167	36	0	0	0	0
	Sex												
	Male	19,299	14,516	4,783	32.9	1,897	72	889	35	0	0	0	0
	Female	8,208	5,968	2,240	37.5	771	82	255	32	0	0	0	0
	Origin												
	Imported	2,671	2,287	384	16.8	58	19	7	3	< 5	1	0	0
	Domestic	24,331	18,272	6,059	33.2	2,454	73	1,140	37	0	0	0	0

EU/EEA: European Union/European Economic Area; PI: prediction interval.

EU/EEA countries with data for 2012–19 included in this analysis are: Austria, Belgium, Cyprus, Czechia, Denmark, Estonia, Finland, France, Greece, Hungary, Ireland, Italy, Latvia, Lithuania, Malta, the Netherlands, Norway, Poland, Portugal, Romania, Slovakia, Slovenia, Spain, Sweden and the United Kingdom.

As presented in Table 2, there is no age group where we did not see cases above the expected levels or above the 80% and 95% PI based on the weekly prediction models. The increase over the expected was between 19.8% and 47.0%. Both cases among males and females as well as imported and domestic cases showed increases. Figures presenting weekly reported number of LD cases by date of onset with retrospective prediction for 2017–19, stratified by age group, sex and importation model are available in Supplementary Material S1–10.

### Sensitivity analysis results on prediction

When excluding cases attributed to the 2014 LD outbreak in Portugal, there was a slightly higher number of cases above the expected levels in 2017–19 (35.4% increase from the prediction). The difference in the number of cases was observed mostly in the last months of 2019, as the excluded outbreak occurred in November 2014 and thus decreased the expected number of cases during that part of the year. Figures and tables presenting weekly reported number of LD cases by date of onset, with retrospective prediction for 2017–19, excluding outbreak cases in Portugal from 2014, are available in Supplementary Material S21–S22.

### Interrupted time series

**Figure 3:**
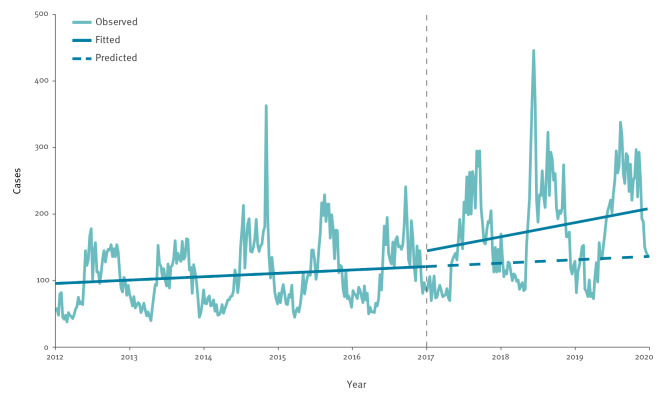
Weekly number of reported Legionnaires’ disease cases by date of onset with interrupted time series trend line, EU/EEA, 2012–2019 (n = 55,821 cases)

The ITS graph in Figure 3 shows the observed LD cases from 2012 to 2019 with a fitted line on cases with removed seasonal component. As shown in Table 3, while the overall weekly number of LD cases did not have a statistically significant trend during 2012–16 (β_1_= 0.044, 95% CI: −0.003 to 0.091), we observed a significant positive change in trend during 2017–19 (β_3_ = 0.135, 95% CI: 0.022–0.243).

EU/EEA: European Union/European Economic Area.

EU/EEA countries with data for 2012–19 included in this analysis are: Austria, Belgium, Cyprus, Czechia, Denmark, Estonia, Finland, France, Greece, Hungary, Ireland, Italy, Latvia, Lithuania, Malta, the Netherlands, Norway, Poland, Portugal, Romania, Slovakia, Slovenia, Spain, Sweden and the United Kingdom.

Residual analysis plots are available in Supplementary Material S24.

**Table 3:** Trend (2012–2016) and trend change (2017–2019) in modelled Legionnaires’ disease cases based on interrupted time series regression, 25 EU/EEA countries, n = 55,821

Model	Trend 2012–16 (β_1_)			Trend change 2017–19 (β_3_)		
	Estimate	95% CI	p value	Estimate	95% CI	p value
Total	0.044	−0.003 to 0.091	0.064	0.135	0.022 to 0.248	0.019
Age group (years)						
< 40	0.001	−0.004 to 0.006	0.749	0.015	0.002 to 0.028	0.025
40–49	0.003	−0.007 to 0.013	0.602	0.014	−0.010 to 0.037	0.247
50–59	0.015	0.001 to 0.029	0.033	0.025	−0.008 to 0.057	0.139
60–69	0.016	0.001 to 0.031	0.036	0.054	0.018 to 0.089	0.004
70–79	0.013	0.002 to 0.023	0.018	0.046	0.021 to 0.072	< 0.001
≥ 80	0.016	0.006 to 0.026	0.002	0.041	0.017 to 0.065	0.001
Sex						
Male	0.030	−0.006 to 0.067	0.098	0.094	0.008 to 0.181	0.033
Female	0.021	0.006 to 0.037	0.008	0.065	0.027 to 0.102	0.001
Origin						
Imported	0.013	0.006 to 0.020	< 0.001	-0.007	−0.024 to 0.009	0.380
Domestic	0.039	−0.005 to 0.083	0.085	0.125	0.019 to 0.232	0.021

CI: confidence interval; EU/EEA: European Union/European Economic Area.

EU/EEA countries with data for 2012–19 included in this analysis are: Austria, Belgium, Cyprus, Czechia, Denmark, Estonia, Finland, France, Greece, Hungary, Ireland, Italy, Latvia, Lithuania, Malta, the Netherlands, Norway, Poland, Portugal, Romania, Slovakia, Slovenia, Spain, Sweden and the United Kingdom.

By examination of age groups, we can see that age 40–49 years had neither significant 2012–16 trends nor significant trend change in 2017–19. All age groups over 50 years had a significant positive trend before 2017, while the three oldest age groups and < 40 years also have significant trend increase in 2017–19 compared with 2012–16. For females, we found a positive trend during 2012–16 and a trend increase in 2017–19, while for males there was no significant trend before 2017 but a trend increase in the years after. Domestic cases saw a trend increase after 2016, while imported cases had a positive trend before 2017 but no trend change after, reflecting the proportional decrease of imported cases in 2018 seen in Table 1. Figures showing weekly number of reported LD cases by date of onset with interrupted time series trend line, EU/EEA, 2012–19 stratified by age group, sex and importation model are available in Supplementary Material S11–20.

## Discussion

Both methods used in this study showed that the increase in LD cases during 2017–19 was unusual compared with the previous 5-year period. While the retrospective prediction indicated which weeks and which periods of the 3 years showed this increase, it did not give us a clear answer about the trend. The interrupted time series analysis showed that there was also a significant increase in the change in trend. The regression results highlighted that the increase in LD cases in 2017 was the start of an increase in comparison to levels observed under 2012–16.

From the model excluding the 2014 outbreak in Portugal, a small but noticeable impact on the excess cases primarily in 2019 was found, meaning there is likely an underestimation of excess cases in the later part of 2019. However, overall results remained similar, and the main conclusions did not change. As the purpose of this analysis was to examine overall trend in frequency rather than establishing outbreak detection thresholds, all data were included in the final models. A few additional clusters or outbreak events were reported by countries during 2012–19 but none were indicated as contributing to more than 40 cases and none of these spiked as significantly within a week as did the aforementioned outbreak in Portugal. Thus, a sensitivity analysis to consider only this specific outbreak was considered appropriate. The clustering in cases in weeks 21–26 of 2018 was considered sporadic as no outbreaks were identified or reported by countries that could explain this [12].

Exclusion of five countries from the study because of reporting incompleteness decreased the data that could contribute to the models and could limit interpretation of the study for the EU/EEA area in general. However, nearly all excluded data were reported by one country (Germany) from which published analyses of seasonality indicate a similar pattern to EU/EEA data for 2005–15 [13] and, thus, any bias is considered minimal. When running the same analysis with a model only for total cases but with Germany included, we obtained nearly identical results for both cases above the expected levels and for 80% and 95% PI (data not shown).

This study did not aim to identify the causal factors of any significant increase, only to measure and describe the distribution of excess cases. Thus, we are unable to identify any specific cause to such a change in trends from 2017. Of note, increasing rates of LD have also been reported in the United States, with incidence rates rising under 2017–18 among other years [14].

The increase observed in 2017–19 is most noticeable in the population that is already most at risk for the disease, generally individuals aged > 50 years old. One explanation for this phenomenon is that these age groups comprise an increasing proportion of the EU/EEA population. Between 2010 and 2020 people aged 65 years and older increased by 3.0 percentage points as a share of the total population in EU/EEA [15]. Though the study indicated an excess and increasing trend in LD for both males and females, the higher annual proportion of males was consistent throughout the period. An increasing number of men survive to older ages [16], which may result in an increase in the number of males at higher risk for LD. Both could contribute to the rise of LD cases in Europe over time, although it is not clear if this can be linked to the increase observed in 2017–19. A Danish study found no evidence that an increase in LD in Denmark during 2015–18 was due to an ageing population [17].

We found an increase in cases over the predicted levels among imported cases in 2017–19, but no trend increase, while domestic cases increased both over the predicted and in trend. This suggests that it is not only cases related to international travel that are the cause of the overall increase in this period. This is in line with tourism statistics in the EU/EEA countries, which indicate a continuous increase in nights spent in tourist accommodation establishments by both domestic guests and international guests under the period 2012–19 [18]. 

Immunocompromised individuals are at higher risk for developing LD [19]. The burden of cancer in Europe continues to be high among adults aged 65 years and older [20]. This may be an additional factor contributing to the excess of cases observed in the older age groups.

Studies have found an association of LD incidence with weather conditions such as warmer temperatures, higher humidity or more rainfall [21]. Studies in Belgium and the Netherlands found significant associations between the number of LD cases and warm and wet weather [22], specifically a sequence of precipitation, followed by high relative humidity and low wind speed [23]. In recent decades, the frequency and intensity of hot extreme weather in Europe has increased [24] with Europe warming faster than the global average [25]. It is possible that weather patterns could play a role in the observed increase in LD cases. By end of 2019, the years 2014, 2015, 2018 and 2019 were among the warmest years in Europe on record [26]. Increases in the number of cases for other climate-sensitive food and waterborne diseases in Europe under 2016–20 are indicated in a report by the European Environmental Agency [27].

The growth of *Legionella* bacteria in engineered water systems is determined by risk factors such as design, nutrient availability and biofilms, water temperature and flow. Building water systems that lack maintenance and management may accumulate risk factors. Energy-saving approaches on system water temperature, as well as some building materials can also favour growth and thus risk [28,29]. These factors could contribute to the observed increase seen over this period but do not likely explain all excess cases.

We are not aware of any systematic changes by countries in the approach to testing or methods used for LD diagnosis during the 2012–19 period that could contribute to an increase. Most cases notified at the European level are reported as diagnosed by urine antigen testing [2-4,12]. While outbreak events of LD raise awareness of risk for infection with *Legionella* and subsequent testing and clinical diagnosis, outbreak events have been regularly detected and reported since the discovery of the disease in the 1970s. Awareness alone does not likely explain the specific increase since 2017 across the EU/EEA.

## Conclusions

This study indicates the increase in LD observed in Europe under 2017–19 is a change in trend to the preceding 5-year period. The distribution of excess cases per week in this period suggests an overall amplification of seasonal trends. Higher numbers of cases than predicted were observed in all study age groups, males and females, as well as non-imported cases. Among age groups, the strongest significant trend under 2017–19 was observed in the age groups >60 years. These findings suggest that LD may become an infectious disease of increasing significance in Europe in coming years, especially when considering an ageing population, climate changes and water resource pressures. The methodological approach could also be used for future analyses to assess the LD trends in the EU/EEA during the COVID-19 pandemic.

## *Note

The United Kingdom was part of the European Union in the years covered by the surveillance data study period.

## Supplementary Data

2227000 Supplement
