# Supplementary material for: Legionnaires’ disease in the EU/EEA*: increasing trend from 2017 to 2019
Source: Euro Surveill. 2023 Mar 16;28(11):2200114. doi: 10.2807/1560-7917.ES.2023.28.11.2200114 (PMC10021471; doi:10.2807/1560-7917.ES.2023.28.11.2200114)

Supplementary material: Legionnaires' disease in Europe – increasing trend in 2017-2019

Disclaimer:

This supplementary material is hosted by *Eurosurveillance* as supporting information alongside the article Legionnaires' disease in Europe – increasing trend in 2017-2019, on behalf of the authors, who remain responsible for the accuracy and appropriateness of the content. The same standards for ethics, copyright, attributions and permissions as for the article apply. Supplements are not edited by *Eurosurveillance* and the journal is not responsible for the maintenance of any links or email addresses provided therein.

Description of content:

These supplementary materials provide additional illustrative figures of model results, sensitivity analyses on predictions and interrupted time series, and residual analysis plots.

Corresponding figure 2 for 2017-2019 predictions by age group, gender and importation model results as shown in table 2 of the article, are available here as supplementary material S1-10.

Corresponding figure 3 for interrupted time series models by age group, gender and importation model as shown in table 3 of the article, are available here in supplementary material S11-20.

Sensitivity analyses considering the exclusion of outbreak data are available here in supplementary material S21-22.

Residual analysis plots for the two models used in figures 2 and 3 are available here in supplementary material S23-24.

Graphs by age group

Supplement S1: Weekly reported number of Legionnaires' disease cases by date of onset (2012-2019) with retrospective prediction of 2017-2019 based on a 2012-2016 model, age group <40, EU/EEA

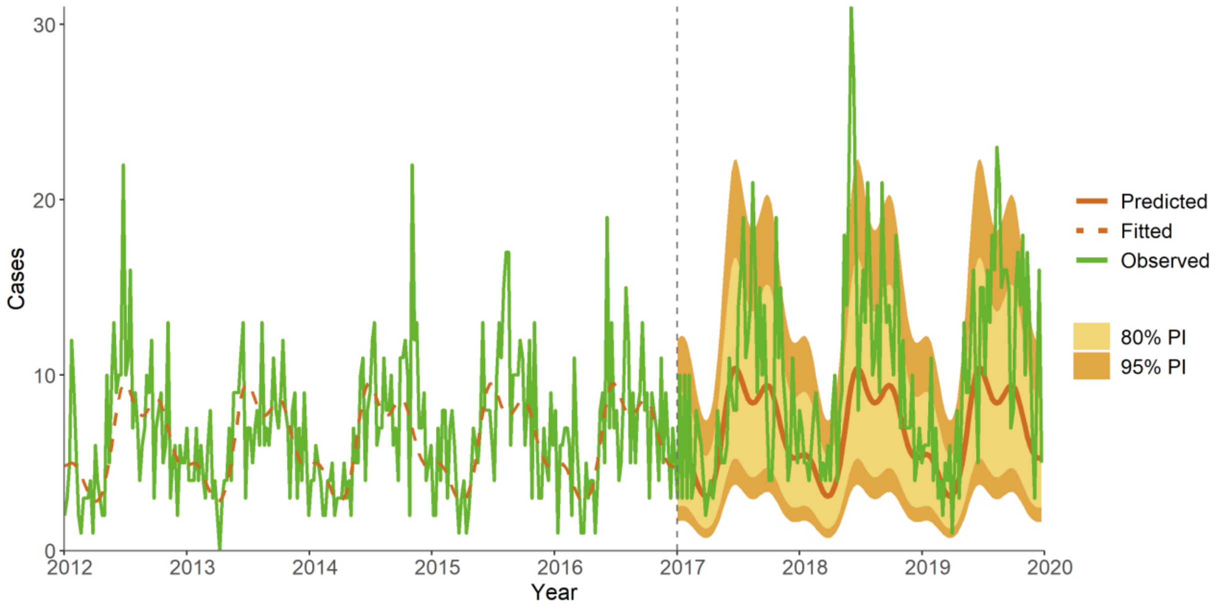

Supplement S2: Weekly reported number of Legionnaires' disease cases by date of onset (2012-2019) with retrospective prediction of 2017-2019 based on a 2012-2016 model, age group 40-49, EU/EEA

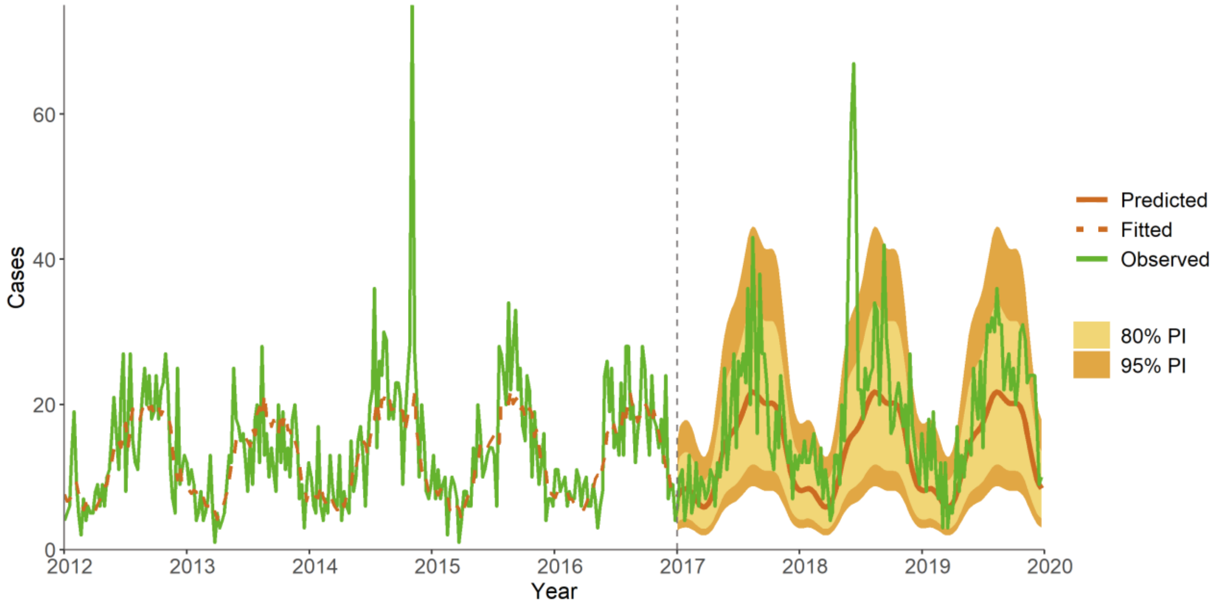

Supplement S3: Weekly reported number of Legionnaires' disease cases by date of onset (2012-2019) with retrospective prediction of 2017-2019 based on a 2012-2016 model, age group 50-59, EU/EEA

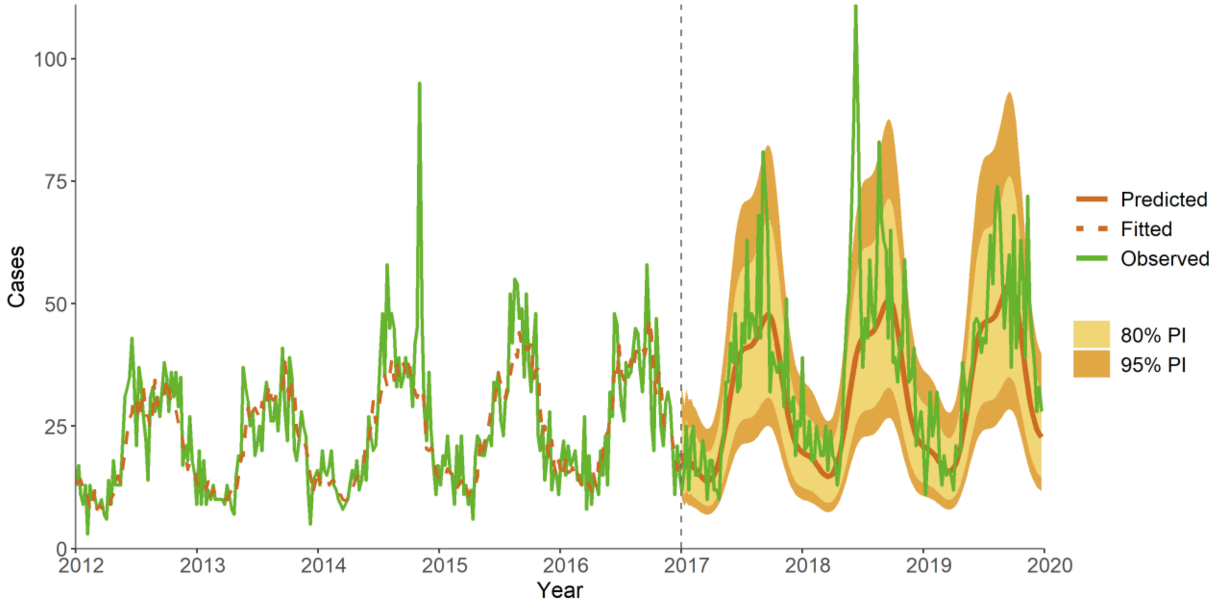

Supplement S4: Weekly reported number of Legionnaires' disease cases by date of onset (2012-2019) with retrospective prediction of 2017-2019 based on a 2012-2016 model, age group 60-69, EU/EEA

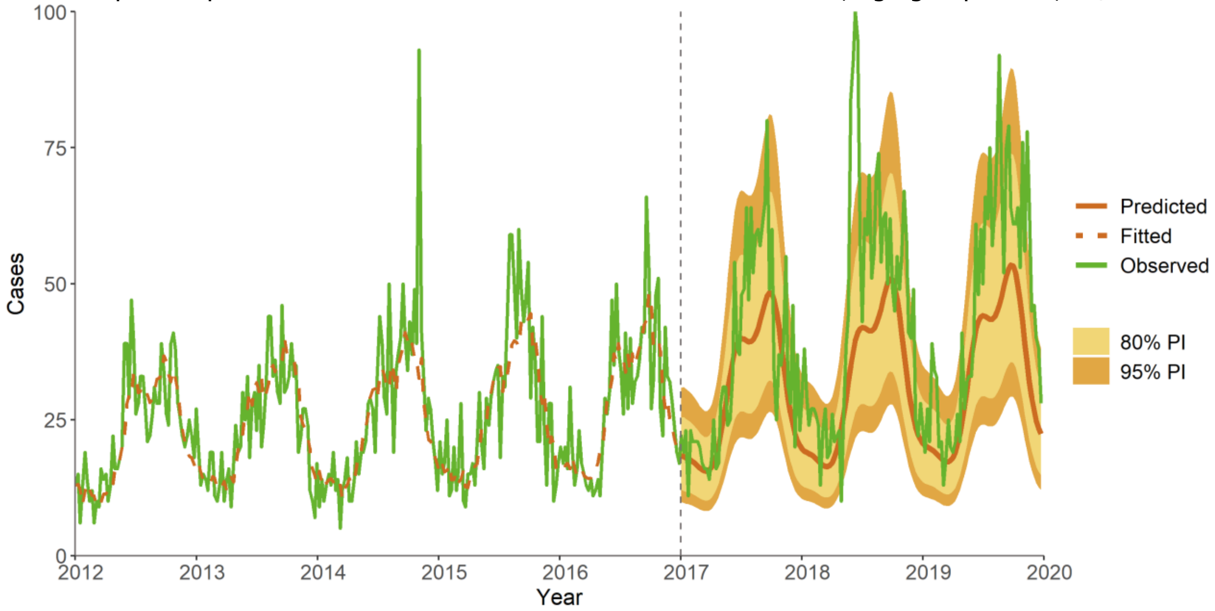

Supplement S5: Weekly reported number of Legionnaires' disease cases by date of onset (2012-2019) with retrospective prediction of 2017-2019 based on a 2012-2016 model, age group 70-79, EU/EEA

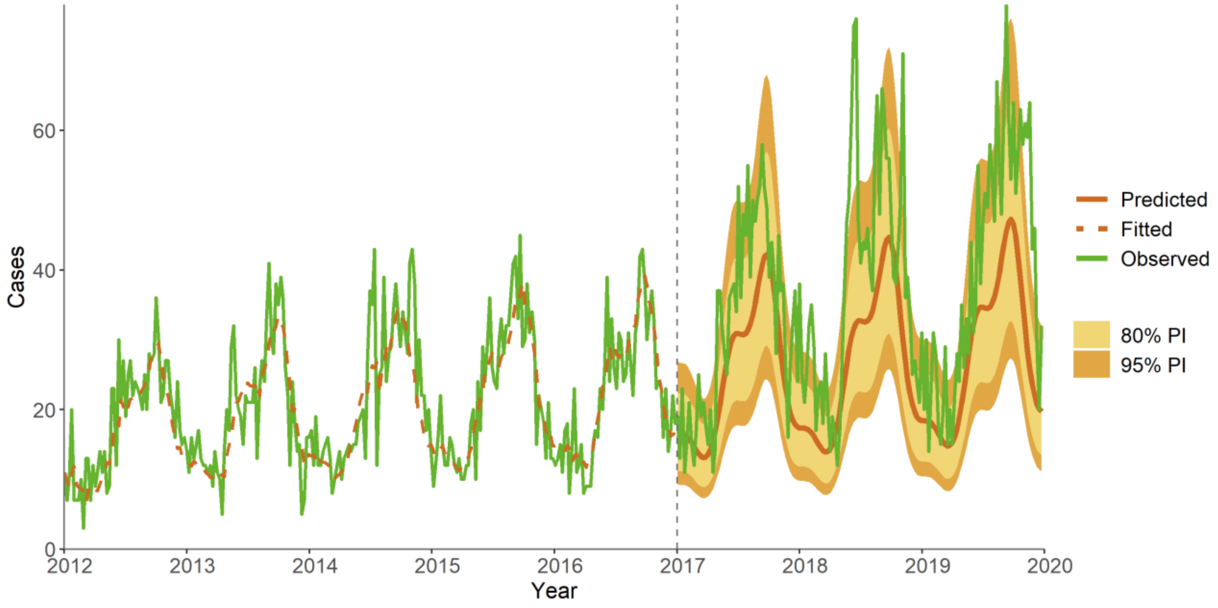

Supplement S6: Weekly reported number of Legionnaires' disease cases by date of onset (2012-2019) with retrospective prediction of 2017-2019 based on a 2012-2016 model, age group 80+, EU/EEA

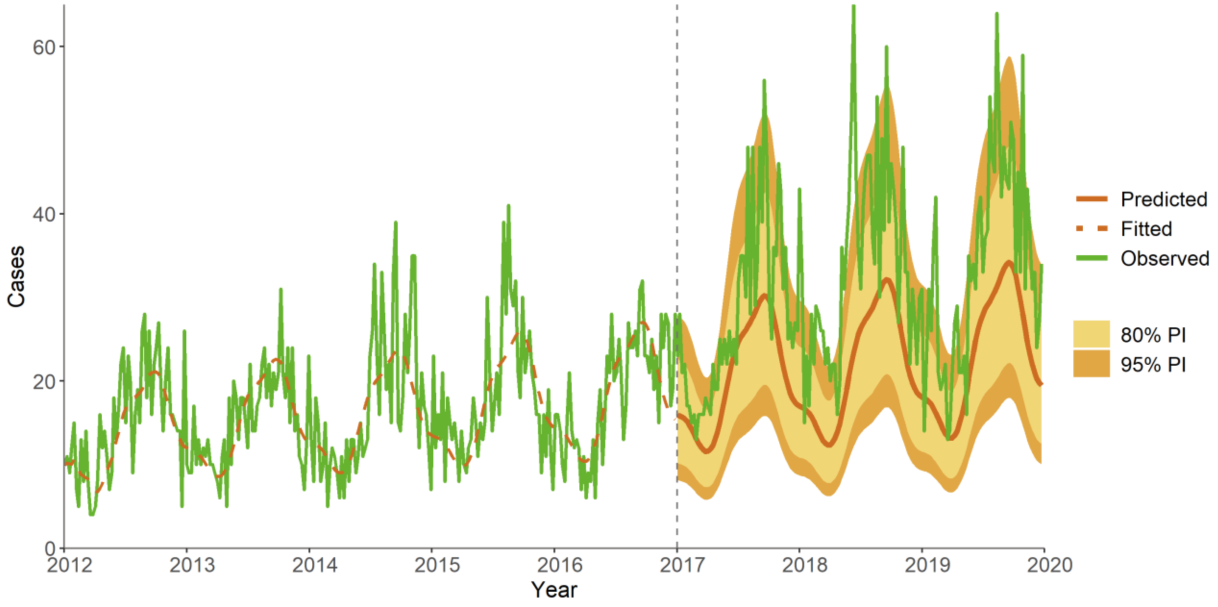

Graphs by gender

Supplement S7: Weekly reported number of Legionnaires' disease cases by date of onset (2012-2019) with retrospective prediction of 2017-2019 based on a 2012-2016 model, males, EU/EEA

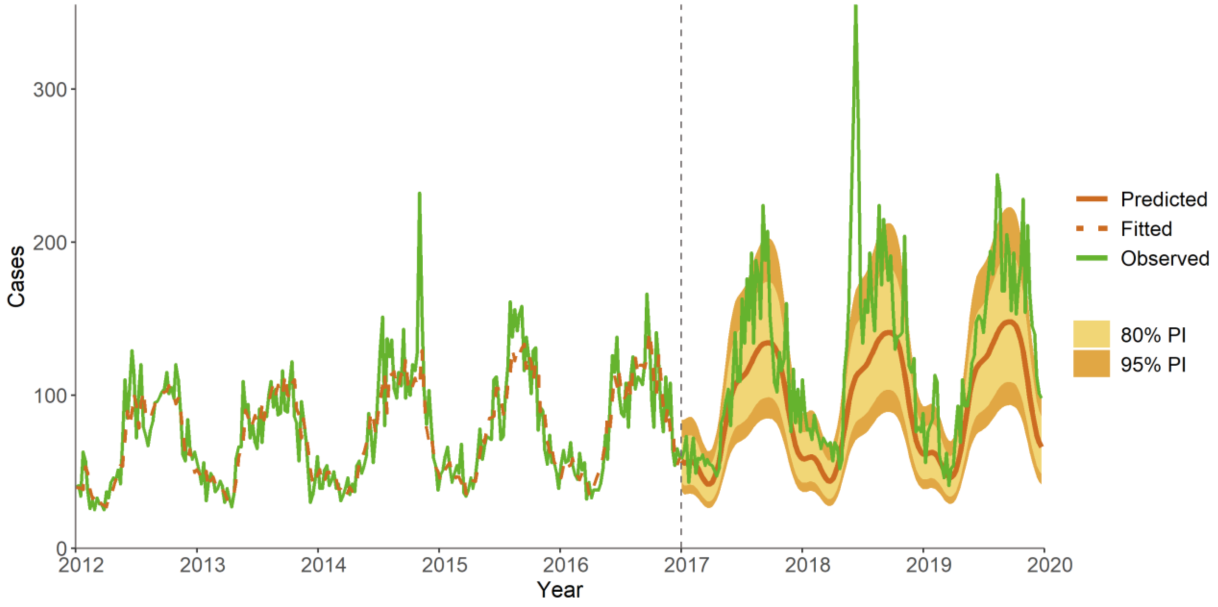

Supplement S8: Weekly reported number of Legionnaires' disease cases by date of onset (2012-2019) with retrospective prediction of 2017-2019 based on a 2012-2016 model, female, EU/EEA

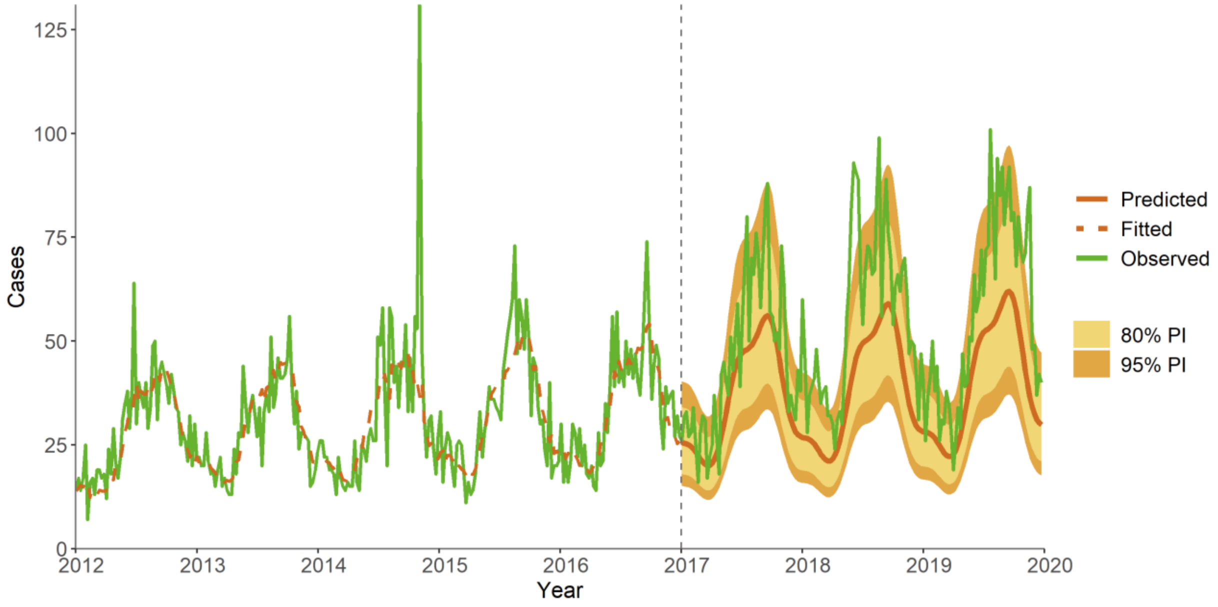

<u>Graphs by imported from abroad</u>

Supplement S9: Weekly reported number of Legionnaires' disease cases by date of onset (2012-2019) with retrospective prediction of 2017-2019 based on a 2012-2016 model, imported, EU/EEA

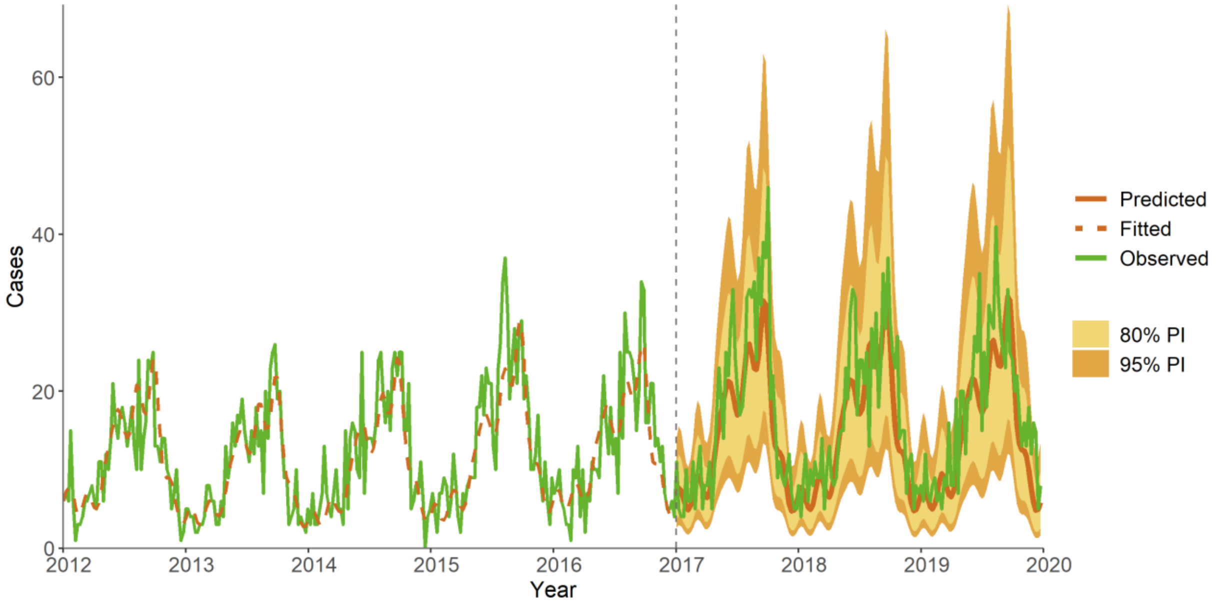

Supplement S10: Weekly reported number of Legionnaires' disease cases by date of onset (2012-2019) with retrospective prediction of 2017-2019 based on a 2012-2016 model, domestic, EU/EEA

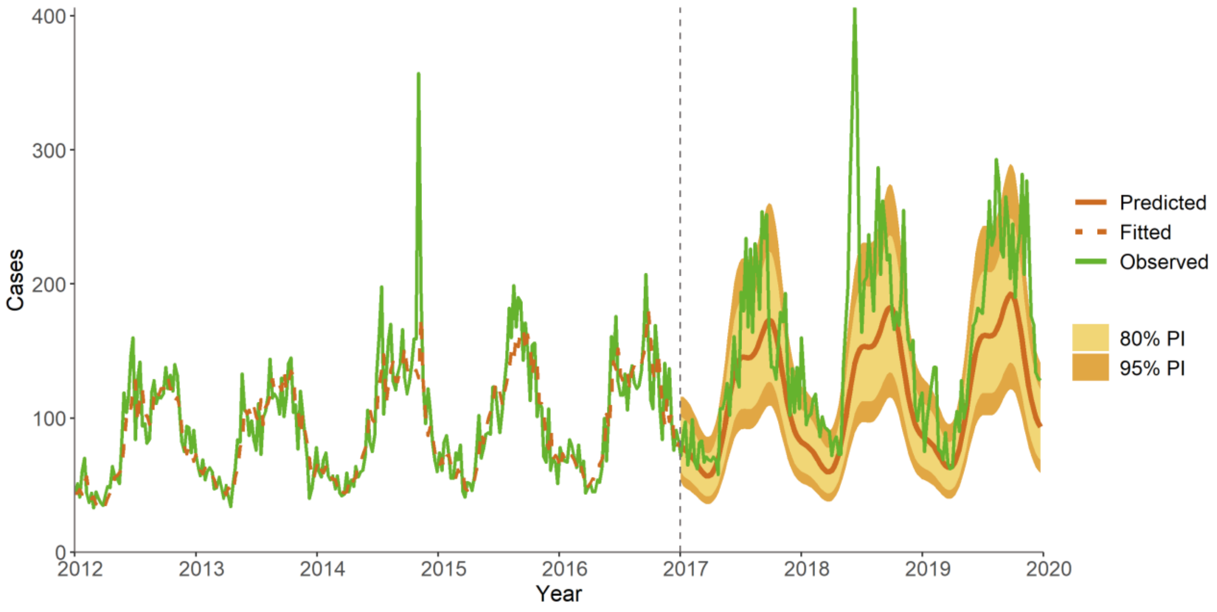

Figures for results discussed in the article: interrupted time series
Graphs by age group

Supplement S11: Weekly reported number of Legionnaires' disease cases by date of onset (2012-2019) with ITS trend line, age group <40, EU/EEA

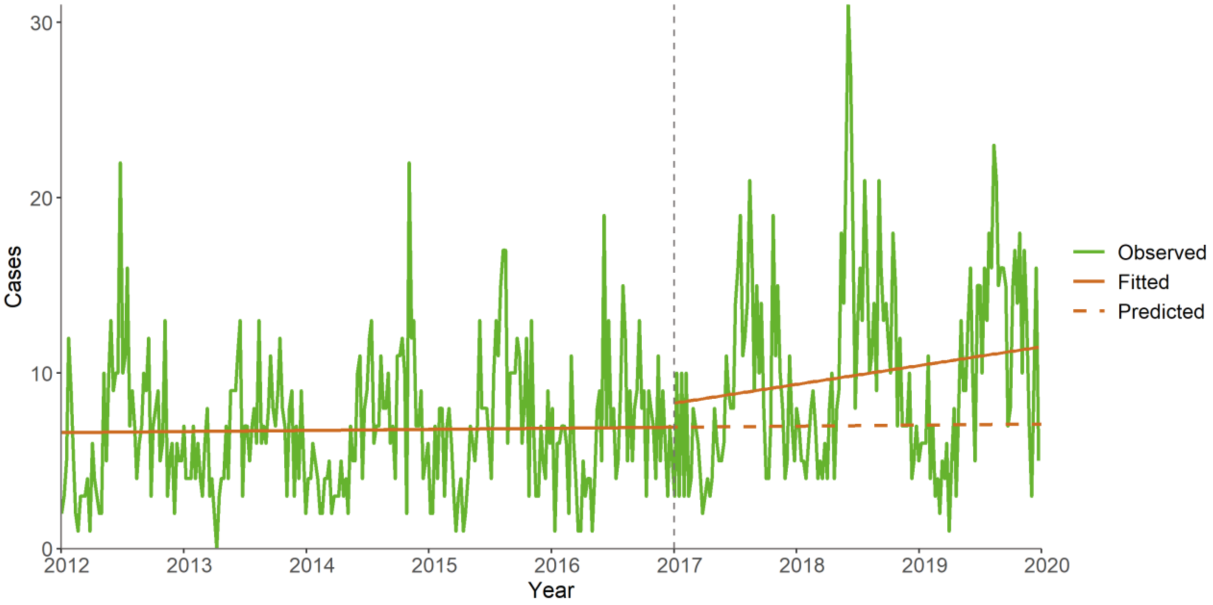

Supplement S12: Weekly reported number of Legionnaires' disease cases by date of onset (2012-2019) with ITS trend line, age group 40-49, EU/EEA

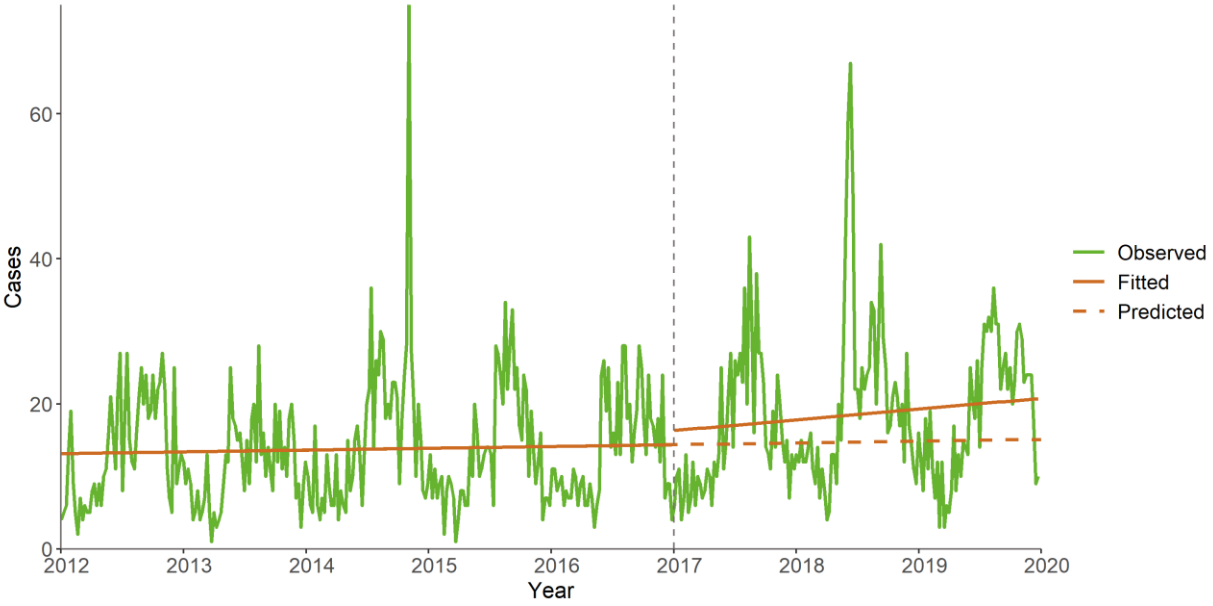

Supplement S13: Weekly reported number of Legionnaires' disease cases by date of onset (2012-2019) with ITS trend line, age group 50-59, EU/EEA

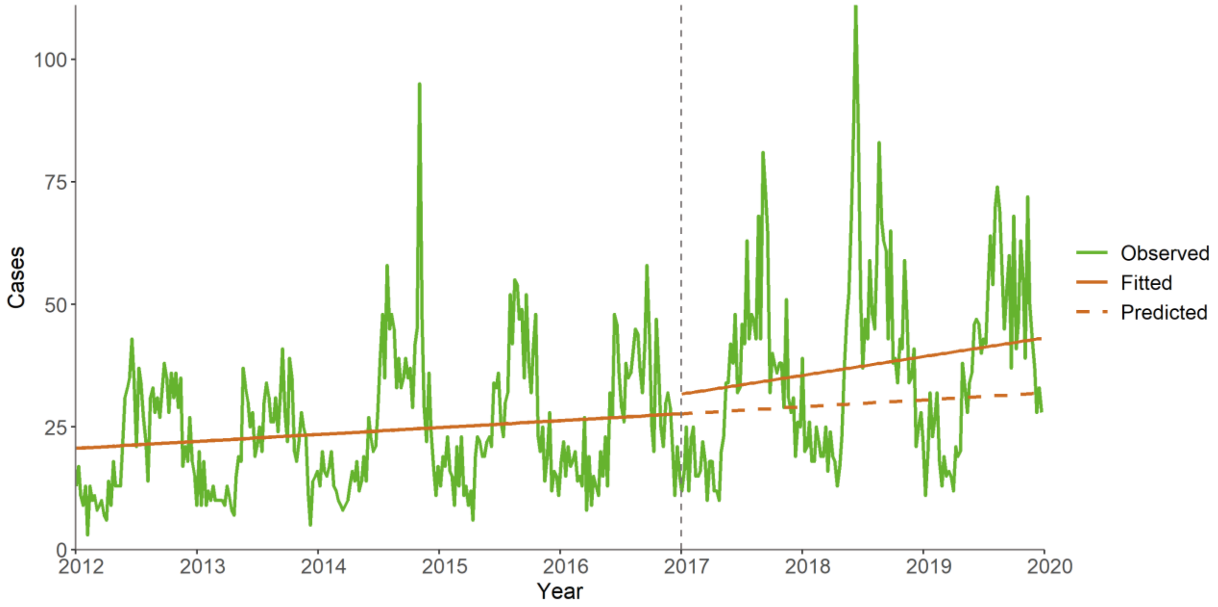

Supplement S14: Weekly reported number of Legionnaires' disease cases by date of onset (2012-2019) with ITS trend line, age group 60-69, EU/EEA

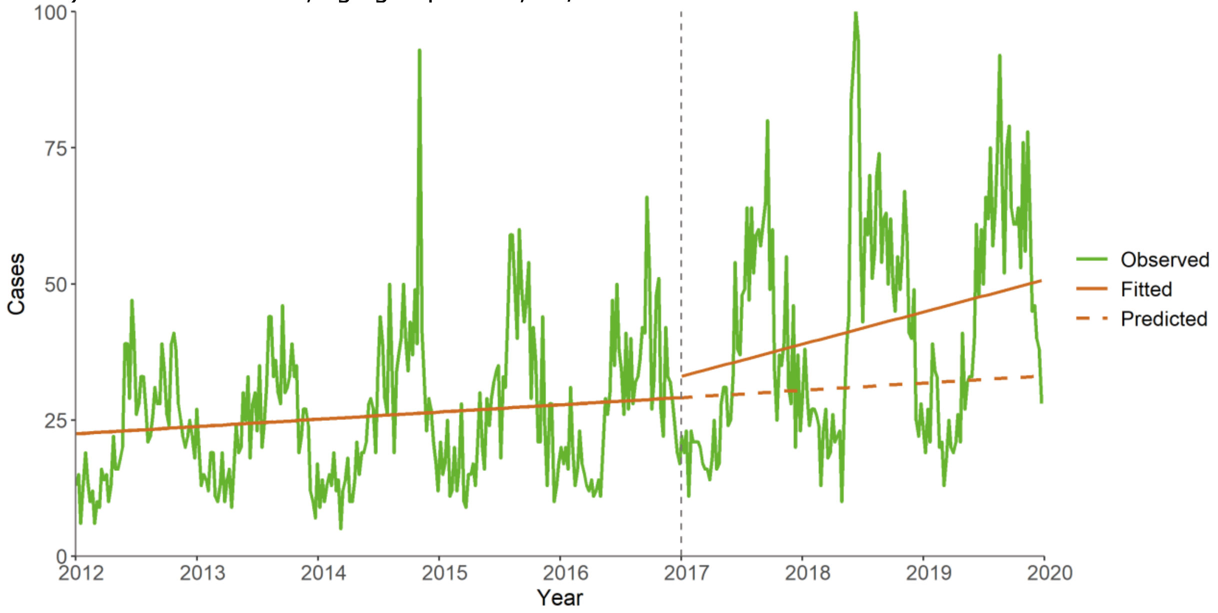

Supplement S15: Weekly reported number of Legionnaires' disease cases by date of onset (2012-2019) with ITS trend line, age group 70-79, EU/EEA

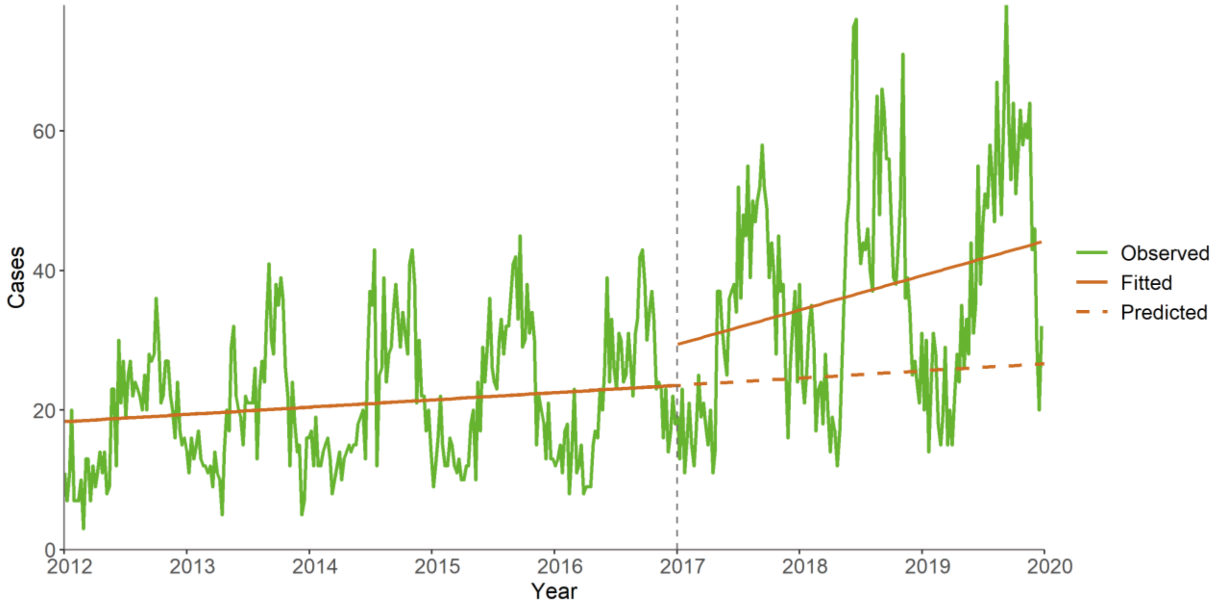

Supplement S16: Weekly reported number of Legionnaires' disease cases by date of onset (2012-2019) with ITS trend line, age group 80+, EU/EEA

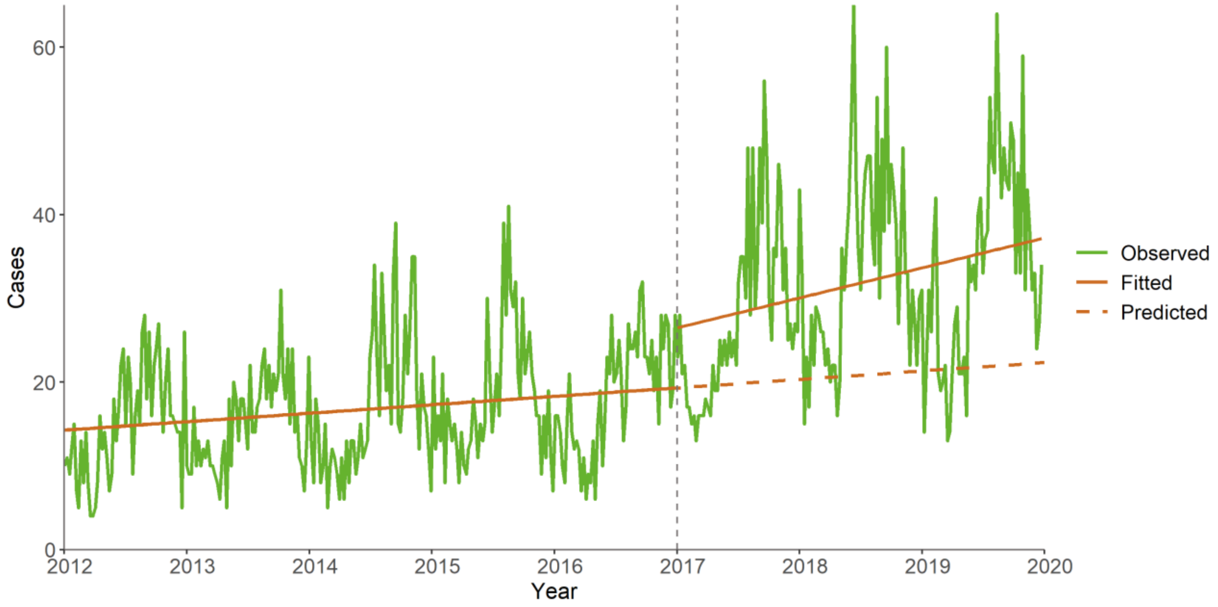

<u>Graphs by gender</u>

Supplement S17: Weekly reported number of Legionnaires' disease cases by date of onset (2012-2019) with ITS trend line, males, EU/EEA

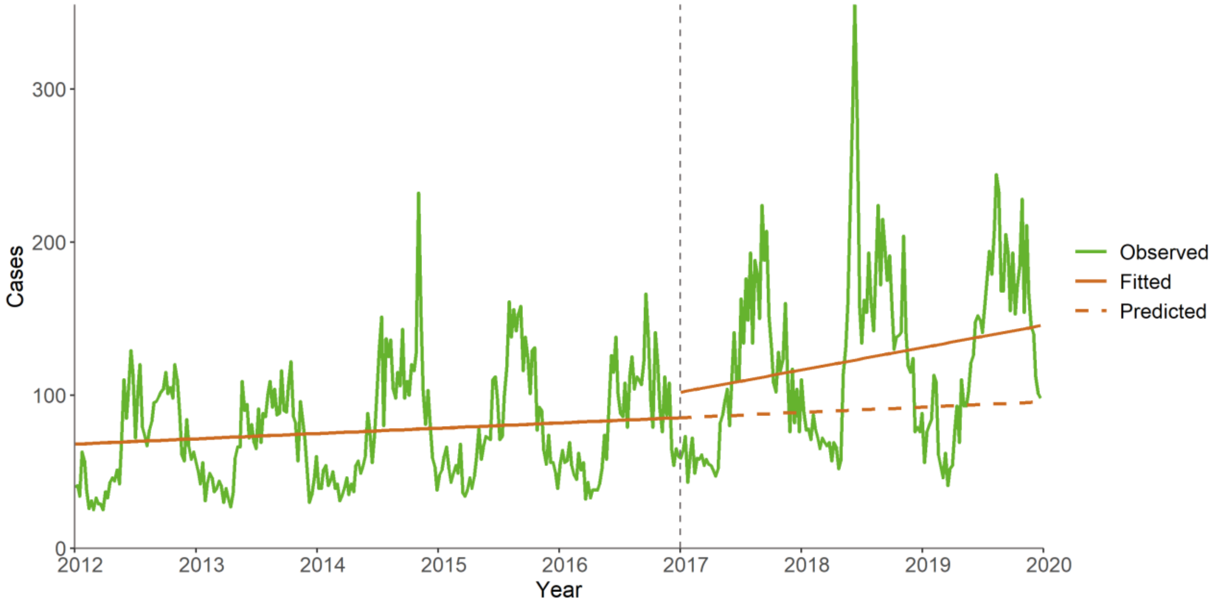

Supplement S18: Weekly reported number of Legionnaires' disease cases by date of onset (2012-2019) with ITS trend line, females, EU/EEA

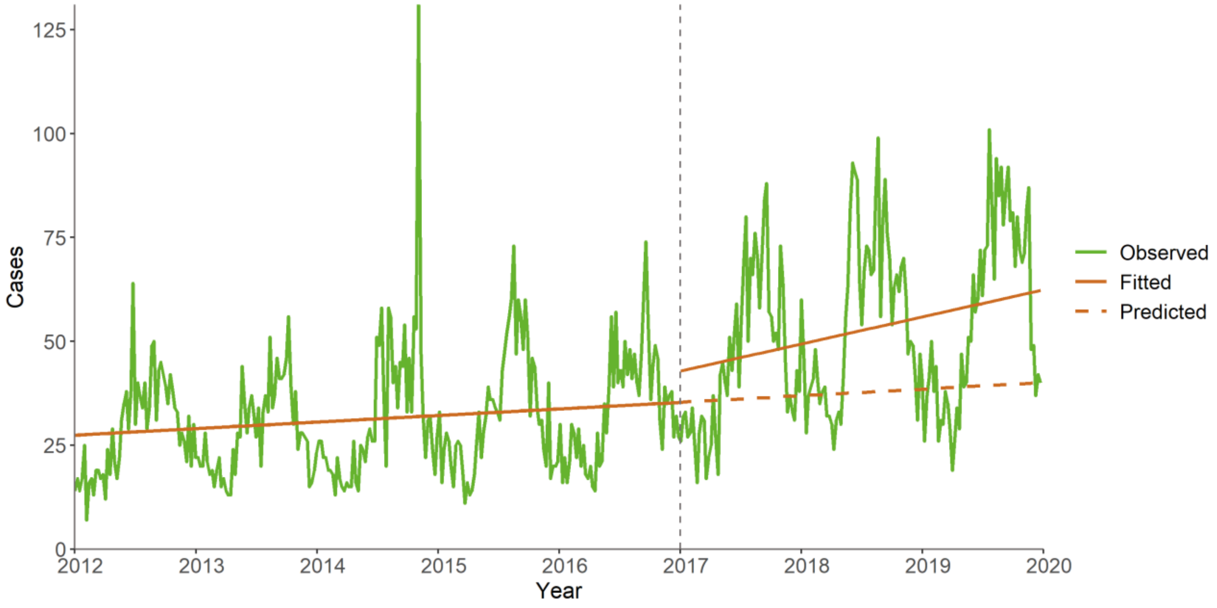

Graphs by imported from abroad

Supplement S19: Weekly reported number of Legionnaires' disease cases by date of onset (2012-2019) with ITS trend line, imported, EU/EEA

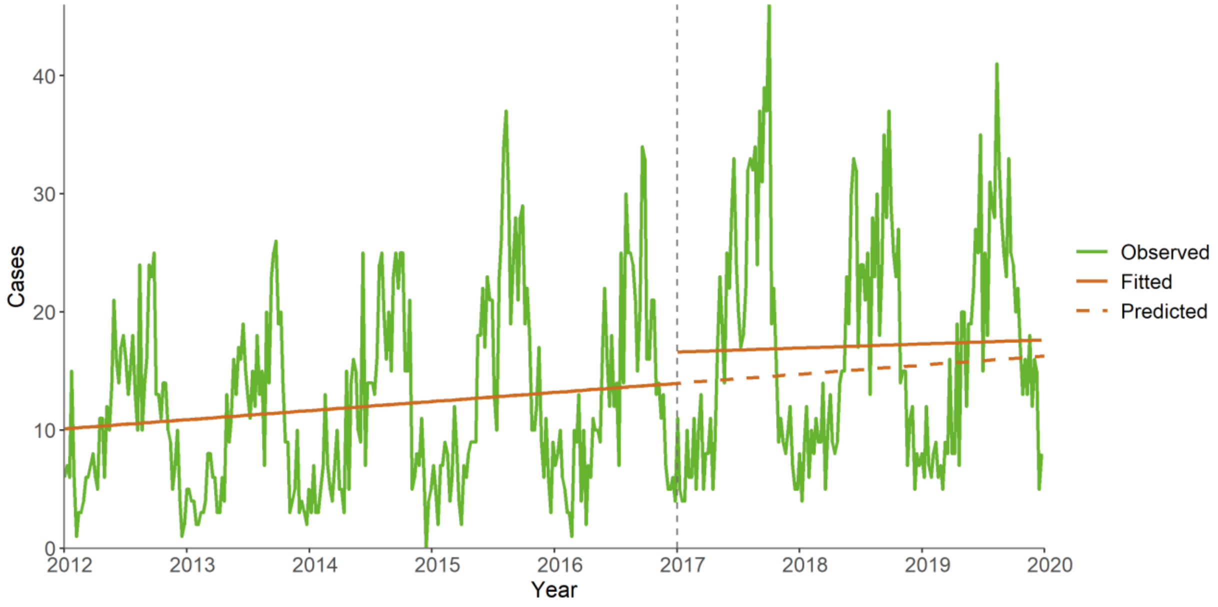

Supplement S20: Weekly reported number of Legionnaires' disease cases by date of onset (2012-2019) with ITS trend line, domestic, EU/EEA

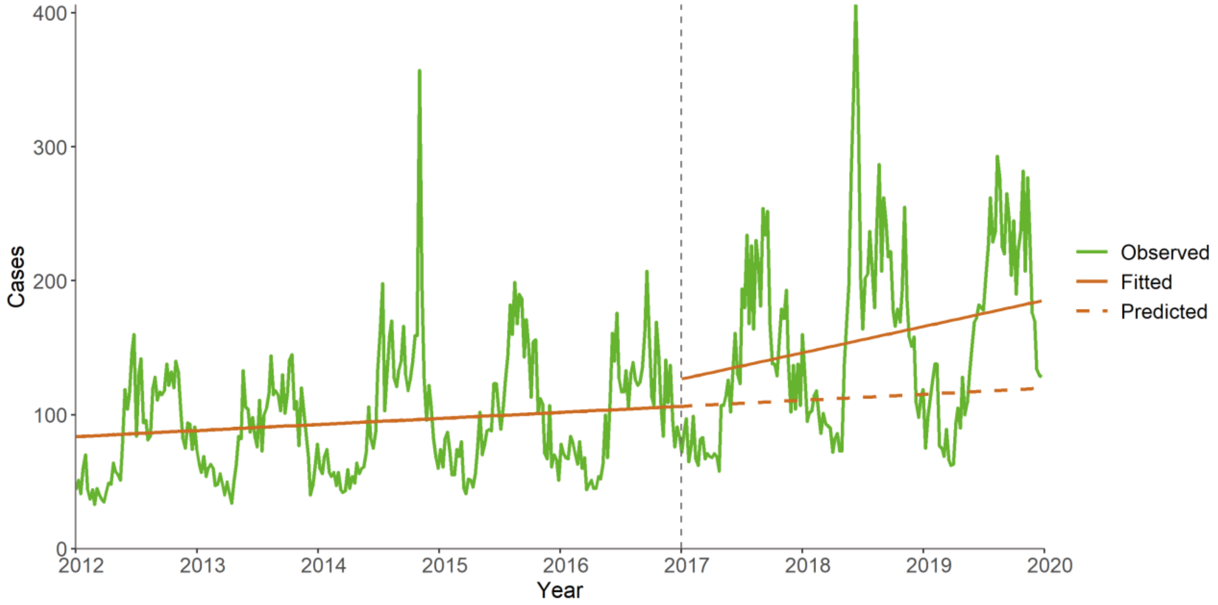

<u>Sensitivity analysis after removing cases from the outbreak in Portugal 2014</u>

Model with and without 291 outbreak cases reported from the outbreak of Legionnaires' disease in Portugal in November 2014.

Supplement S21: Weekly reported number of Legionnaires' disease cases by date of onset (2012-2019) with the retrospective prediction of 2017-2019 based on a 2012-2015 model (excluding the outbreak in Portugal 2014), EU/EEA

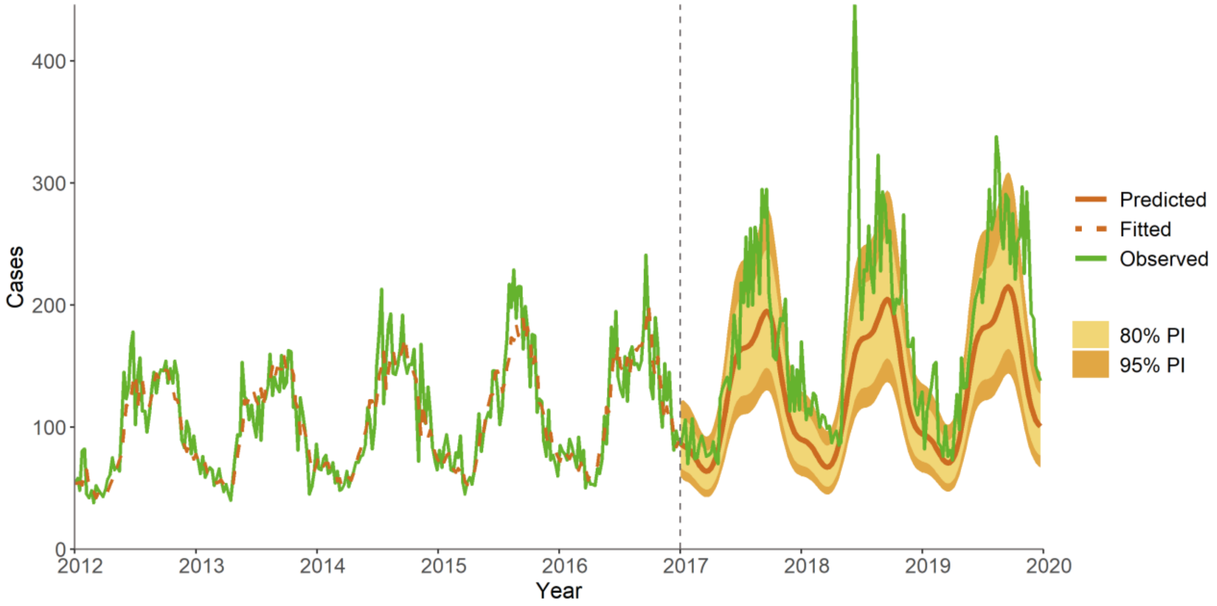

Supplement S22: Annual observed, expected, and excess Legionnaires' disease cases in 2017-2019 based on 2012-2016 weekly retrospective prediction models (excluding the outbreak in Portugal 2014), EU/EEA

Year	Observed cases (O)	Predicted cases (P)	Above prediction		Above 80% Prediction interval		Above 95% Prediction interval	
			Number of cases (O-P)	Percentage (%)	Number of cases	Number of weeks	Number of cases	Number of weeks
2017	7,935	6,445	1,490	23.1	482	22	138	8
2018	9,854	6,771	3,083	45.5	1,588	37	957	24
2019	9,741	7,114	2,627	36.9	1,109	31	524	16
Total	27,530	20,330	7,200	35.4	3,179	90	1,620	48

Supplement S23: Residuals, Autocorrelation function (ACF) plot and histogram for the harmonic regression models with Fourier terms and ARIMA errors (used in Figure 2)

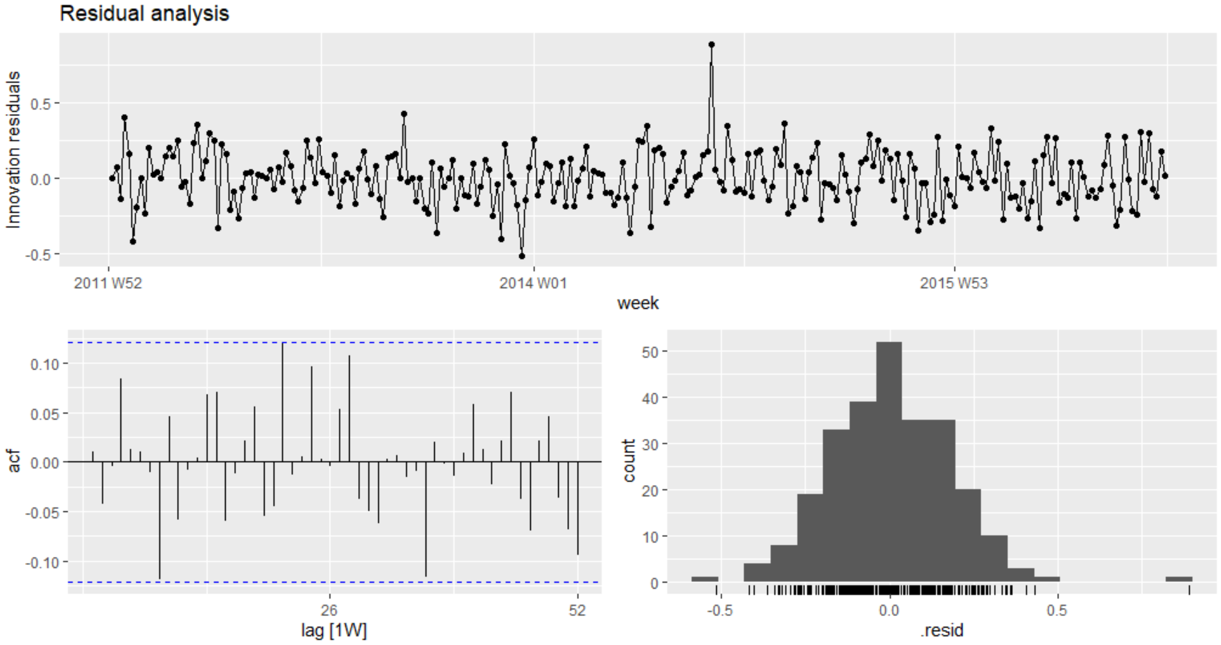

Supplement S24: Residuals, Autocorrelation function (ACF) plot and histogram for the Interrupted Time Series model (used in Figure 3)

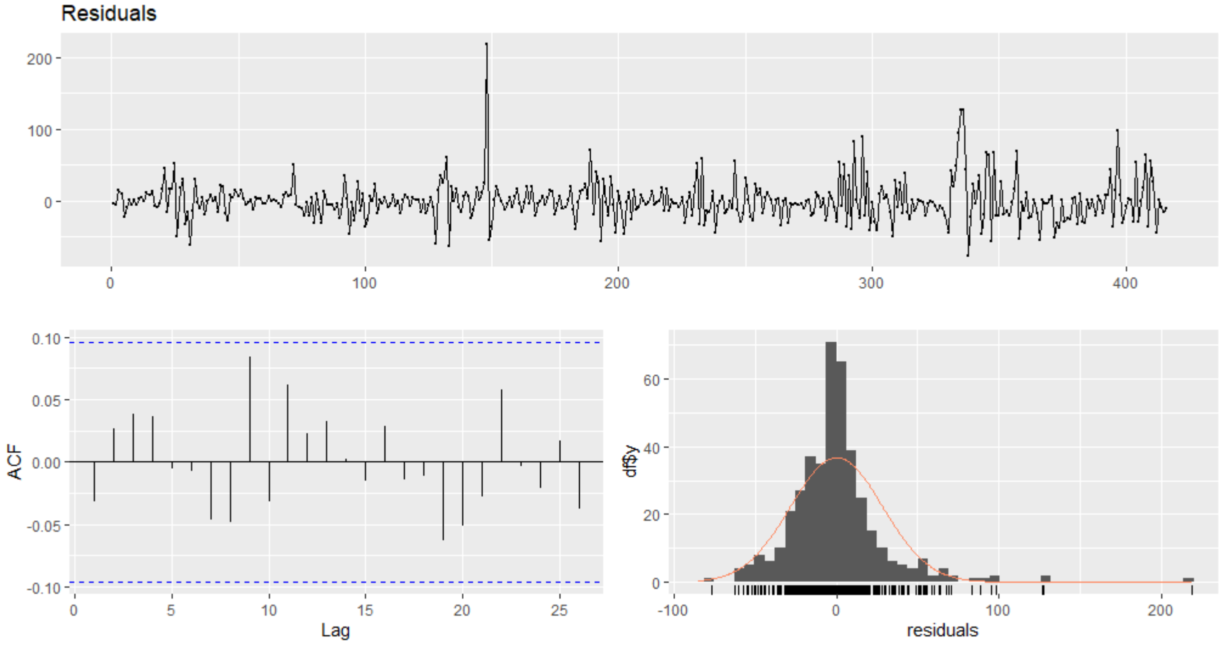

Supplement: Supplement [file 22-00114_PAYNE_SUPPLEMENT.pdf]
